# Social workers’ involvement in advance care planning: a systematic narrative review

**DOI:** 10.1186/s12904-017-0218-8

**Published:** 2017-07-10

**Authors:** Chong-Wen Wang, Cecilia L. W. Chan, Amy Y. M. Chow

**Affiliations:** 1 Jockey Club End-of-Life Community Care Project, Faculty of Social Sciences, The University of Hong Kong, Hong Kong SAR, China; 2 Centre on Behavioral Health, Faculty of Social Sciences, The University of Hong Kong, Hong Kong SAR, China; 3 Department of Social Work and Social Administration, Faculty of Social Sciences, The University of Hong Kong, Hong Kong SAR, China

**Keywords:** Advance care planning, Advance directive, End-of-life care, Social work, Systematic review

## Abstract

**Background:**

Advance care planning is a process of discussion that enables competent adults to express their wishes about end-of-life care through periods of decisional incapacity. Although a number of studies have documented social workers’ attitudes toward, knowledge about, and involvement in advance care planning, the information is fragmented. The purpose of this review was to provide a narrative synthesis of evidence on social workers’ perspectives and experiences regarding implementation of advance care planning.

**Methods:**

Six databases were searched for peer-reviewed research papers from their respective inception through December 2016. All of the resulting studies relevant to both advance care planning and social worker were examined. The findings of relevant studies were synthesized thematically.

**Results:**

Thirty-one articles met the eligibility criteria. Six research themes were identified: social workers’ attitudes toward advance care planning; social workers’ knowledge, education and training regarding advance care planning; social workers’ involvement in advance care planning; social workers’ perceptions of their roles; ethical issues relevant to advance care planning; and the effect of social work intervention on advance care planning engagement. The findings suggest that there is a consensus among social workers that advance care planning is their duty and responsibility and that social workers play an important role in promoting and implementing advance care planning through an array of activities.

**Conclusions:**

This study provides useful knowledge for implementing advance care planning through illustrating social workers’ perspectives and experiences. Further studies are warranted to understand the complexity inherent in social workers’ involvement in advance care planning for different life-limiting illnesses or within different socio-cultural contexts.

## Background

With population aging and extended life expectancy, end-of-life (EoL) care is becoming increasingly a public health or health system problem [1, 2]. Given the fact that terminally-ill persons not only suffer from physical problems but also face the problems associated with psychological, social, spiritual, and financial concerns, an interdisciplinary care approach is often applied. Social workers in varied care settings are often the key professionals who interface with patients and their families during life transitions because of their skills of communication, negotiation, support and advocacy [3]. Social work practice is founded on a holistic model which embraces all areas of need [4]. The involvement of social workers is critical to EoL care provision [5–7].

Generally, social workers work with dying persons and their families in three major aspects: they are called upon to address psychosocial and spiritual concerns of dying persons and their families, to help the dying persons make advance care planning (ACP) or formulate advance directives (ADs), and to provide grief counselling for pre-bereaved or bereaved family members [6]. Usually, social workers are well-trained or educated in the areas of psycho-socio-spiritual intervention and grief counseling, even if they are not involved in EoL care practice. What are less presented in social work curriculum in most countries and thus remain uncertain for many social workers may be ACP procedures and associated issues, although educational programs on death and dying or EoL care in general may be provided to them [4, 5, 8, 9].

ACP is a voluntary process of discussion that extends the rights of competent adults and enables them to express or communicate their wishes about future health care through periods of decisional incapacity [3, 10, 11]. During this process, ADs may or may not be formulated, which are “a person’s verbal or written expression or instructions about his or her wishes, preferences, or plans for future medical treatments or health care, in the event that he/she becomes unable to communicate” [3, 10]. The role of ADs is to enable health care professionals to legally or ethically ascertain patients’ preferences for care, so as to protect their rights and promote their quality of life and quality of death. Common elements of ACP or ADs include living wills, health care proxy (HCP) or durable power of attorney, refusal to unwanted invasive treatments, preferred priority of care, and preferred place of care. ACP allows patients to retain control over any life-prolonging treatments they may receive in the situation that they are incapable to speak for themselves [12]. The effectiveness of ACP has been studied among various older people at different care settings using different outcome measures. There is evidence that ACP positively impacts the quality of EoL care. A systematic review of 113 studies suggested that ACP interventions decreased life-sustaining treatment (LST), increased use of hospice and palliative care, prevented hospitalization, and increased compliance with patients’ end-of-life wishes [13]. Another systematic review indicated that ACP decreased hospitalization rate of nursing home residents by 9–26% and increased the number of residents dying in their nursing homes by 29–40% [14]. Moreover, ACP increased the completion of ADs, concordance between preferences for care and delivered care, and likelihood of improvement of other outcomes for patients and their loved ones [15].

ACP throughout the end of life is an important facet of professional social work practice with older patients and their families, since social workers have a greater degree of familiarity with their clients’ wishes and needs than other health professionals [3, 16, 17]. Moreover, social work is committed to respecting, valuing, and empowering patients [4]. A number of studies have documented social workers’ attitudes toward, knowledge of, and involvement in ACP. However, the information is fragmented. To date, the literature lacks a systematic review of the findings of relevant studies in this field. To inform evidence-based social work practice, professional social work education, and healthcare or social care policy making, as well as to identify areas for future scientific studies, an examination of the findings generated from empirical or scientific research with regard to social workers’ contribution in this area seems necessary. Given that EoL care is being included in the global health agenda [18], such effort may have important implications for the development of social work practice in the delivery of quality EoL care in those countries where EoL care services are underdeveloped. Thus, the purpose of this review was to provide a systematic narrative synthesis of the findings reported in peer-reviewed publications that examined social workers’ perspectives and experiences regarding the implementation of ACP for older persons, so as to better understand social workers’ contribution in this field and the process of how ACP was conducted in social work practice.

## Methods

### Eligibility criteria

The following criteria were used for study selection. (1) Types of studies. Original studies with any study design, except case reports, were considered. That is, both quantitative and qualitative studies, both descriptive and interventional studies, both cross-sectional and longitudinal studies, and both controlled and uncontrolled studies were eligible for inclusion. In order to provide a degree of quality control in study selection, only the studies published in peer-reviewed journals or unpublished theses that had been examined by reviewers were included. Conference proceedings and the publications that were not data-driven, such as editorials, commentaries, literature reviews, and discussion documents, were excluded. (2) Types of participants. Studies that included social workers either as a whole sample or as a subsample were included. Studies that included a mixed sample of health care professionals but did not make a comparison between social workers and other care professionals were excluded. Studies focusing on dying persons, caregivers, case managers and care professionals other than social workers were excluded. (3) Types of outcomes. Studies of ACP or ADs were included. Studies of EoL care in general rather than ACP in particular were excluded. Studies of concurrently medical decision making, psychiatric advance directives, or pediatric advance care planning were excluded.

### The literature search

The following electronic databases were searched from their respective inception through December 2016: PubMed/Medline, Web of Science, AMED, CINAHL, SocINDEX, and PsychINFO. The following terms were used with such a search string: (advance care planning or advance directive* or advance care directive* or advance statement* or end of life care planning or end of life planning or end of life decision making or do-not-resuscitate order* or life-sustaining treatment or living wills or health care proxy or health care surrogate*) and (social work or social worker* or social services staff or social services professional* or social care staff or social care professional* or social care provider*). We searched the electronic databases for articles containing these terms in the title, abstract or keywords. No limits were imposed on language. The reference lists of all included studies and other archives of the located publications were hand-searched for further relevant articles.

### Data extraction and synthesis

All records generated through the searches were exported into EndNote. The titles and abstracts were reviewed manually. Irrelevant records were excluded according to the eligibility criteria. If a record was potentially eligible for inclusion, the full-text was retrieved for further screening. Study selection, data extraction and data synthesis were conducted by one main researcher (CW) and then verified by other researchers (AC, CC). Any discrepancies were resolved by discussion. From each of the included studies, we extracted the following information onto a customized data-extraction sheet: research objective, type of study design, type of participants, sample size, and major findings. We classified the included studies into different categories according to the study design and participants. A thematic analysis or synthesis of major findings of the included studies was then performed. For the studies that had multiple themes, they were allocated into multiple groups. Where uncertainty existed, the full-text of the article was reexamined.

## Results

**Fig. 1:**
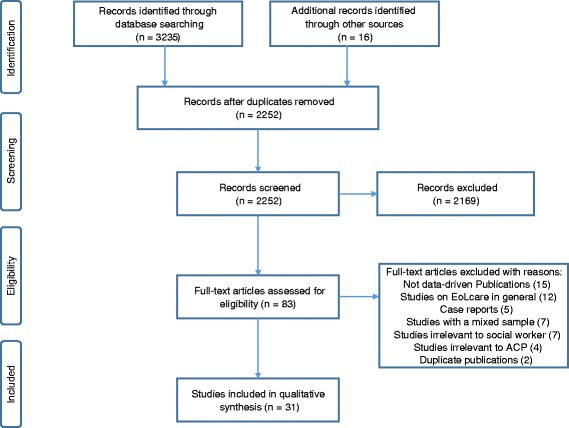
Flow diagram

Our searches identified 2252 potentially relevant records, and 2169 records were removed after screening the titles and abstracts. Full reports of 83 publications were acquired and 52 papers were further excluded as they were not data-driven publications, studies on EoL care in general, studies with a mixed sample, studies irrelevant to either social worker or ACP, case reports, and duplicates. Consequently, 31 articles published between 1994 and 2016 met the eligibility criteria (Fig. 1).

Of the included studies, twenty-six were conducted in the US and the remaining five were conducted in South Korea [19, 20], Singapore [21], and Israel [22, 23], respectively. Twenty studies used cross-sectional surveys, three used qualitative interviews [24–26], and the remaining eight were interventional studies [27–34]. Sixteen descriptive studies included a sample of only social workers [11, 19, 20, 22, 24, 25, 35–44]. Of them, four included social workers recruited from hospitals, additional four included social workers from nursing homes, two included social worker students, and the remaining six included a mixed sample of social workers (Table 1). Seven descriptive studies [21, 23, 26, 45–48] included a mixed sample of multidisciplinary care professionals, in which social workers were compared with nurses and/or physicians (Table 2). In the eight interventional studies, including three randomized controlled trials, one retrospective cohort study, one quasi-experimental study and three uncontrolled studies, the ACP interventions were implemented or facilitated by social workers, whilst the patients or residents were employed for outcome assessment (Table 3). The participants included adult orthopedic surgical patients who were admitted to a hospital for hip or knee replacement surgery, veteran patients recruited at different settings, newly admitted long-term care residents, outpatients, and homeless persons. Sample sizes in the included quantitative studies ranged from 10 to 13,913, with a median of 171. Sample sizes in the included qualitative studies ranged from 11 to 15.

**Table 1:** Summary of descriptive studies that included a sample of only social workers

Study (country)	Objective	Design	Participants	N	Major findings
Han, 2016 [20] (South Korea)	To examine social workers’ understanding of a patient’s right to EoL care decisions in long-term care facilities	A cross-sectional survey	A non-probability sample of social workers in long-term care facilities.	297	1. 55.3% of the respondents had experience aiding elderly patients and/or family members by providing information about ADs. 2. Social workers’ understanding of a patient’s right to EoL care decisions was associated with their general and medical knowledge of ADs, experience in EOL care, and relevant training.
Kwon & Kolomer, 2016 [19] (South Korea)	To examine social workers’ awareness & attitudes toward EoL care planning	A cross-sectional survey	A non-probability sample of gerontological or geriatric social workers	246	1. 73% of the social workers reported no knowledge of ADs. 2. 22% had education or training in EOL issues. 3. 83.3% reported that they did not have any experience working with terminally ill patients. 4. 41.1% felt comfortable with death discussion in general, and 26.8% felt comfortable in having a discussion about death with older clients. 5. Social workers who emphasized self-determination, professed a preference for hospice care, and were comfortable discussing death were more likely to have a positive attitude to ACP.
Stein et al., 2016 [44] (USA)	To explore social worker involvement and leadership in ACP conversations with patients and families	A cross-sectional, web-based survey	Social workers employed in hospice, palliative care, and related settings	641	1. 96% of the respondents reported that social workers in their department conducted ACP discussions with patients/families. 2. 80% of the respondents reported that social workers were responsible for educating patients and/or families about ACP options. 3. 68% of the respondents reported that social workers were responsible for documenting ACP. 4. Compared with those at other care settings, oncology and inpatient palliative care social workers were less likely to be responsible for ensuring that patients/families are informed of ACP options and documenting ACP preferences.
Kwon et al., 2014 [11] (USA)	To examine the attitudes of social work students toward EoL care planning,	A cross-sectional survey	A cluster sample of social work students at a school	102	1. 72% of the participants indicated that they felt comfortable discussing the topic of death. 2. 97% indicated that self-determination is a very important principle in social work practice. 3. 75% answered that they would be troubled if problems of self-determination resulted in conflicts. 4. Positive attitudes toward ACP were associated with higher levels of comfort when discussing death, more emphasis on self-determination, and apprehension of conflicts of self-determination.
Sherwood, 2012 [24] (USA)	To explorer the knowledge and attitudes of social service workers’ in nursing homes regarding the preparation of ADs.	Qualitative interviews	A purposive sample of 15 social work assistants came from 11 facilities.	15	1. 60% of the respondents said that they receive no training regarding ADs, although 53% said they have recently received training on the POLST. 2. One third of the respondents stated that social workers initiate the AD conversation, 33% said that nurses initiate the AD conversation, and others said that AD conversation is done at admission or during care planning meeting with family members. 3. 73% stated that they have conversations about AD during care meetings. 4. 40% indicated that they provide a facility-specific form to residents regarding ADs, and additional 40% noted that no AD forms are provided at all. 5. All respondents indicated a situation they had once dealt with where the family requests something different from what is written on the patient’s ADs.
Gutheil & Heyman, 2011 [35] (USA)	To examine social workers’ attitudes toward EoL planning	A cross-sectional survey	Randomly selected social workers (response rate: 42%)	844	1. Social workers in health and aging had significantly higher positive attitude scores than those not in health and aging. 2. For social workers in health and aging, 72.3% had completed a HCP, compared with 48.8% in other than health and aging. 3. For social workers in health and aging, 88.8% had training in EoL care issues, compared with 53.2% in other than health and aging. 4. For social workers in health and aging, 78.3% indicated that they received EoL care training in continuing education programs.
Peck, 2009 [38] (USA)	To examine the extent to which the death anxiety of oncology social workers impacts the completion of personal ADs, and their communication about ADs with patients.	A cross-sectional survey	A random sample of oncology social workers (response rate: 25%)	114	1. 68% of the respondents reported having completed a personal AD. 2. Motivating factors for personal AD completion included professional experience, peace of mind, control over decisions, and the desire to not have family members make decisions. 3. As death anxiety scores increased, the communication scores decreased related to disclosure of information about ADs (to patients) and values in living. 4. There was no significant difference in death anxiety scores between social workers who had completed and those who had not completed an AD.
Heyman & Gutheil, 2006 [39] (USA)	To examine the factors associated with social workers’ involvement in EoL planning.	A cross-sectional survey	A random sample of NASW members in health and aging (response rate: 63%)	390	1. 62% of the respondents stated they had a role in EoL planning. 2. 72.5% rated education as an important role and 31.5% said that facilitating decision making was an important role for them in EoL planning. 3. 75% felt that physicians saw social workers as having a role in work with EoL planning. 4. 44% stated they are very often involved in discussing the HCP with clients, and 37.3% stated they are very often involved in counseling clients regarding HCPs. 5. Almost 25% stated that they were very often involved in receiving requests from other professionals to explain HCP, and 23.8% stated they were very often involved in completing HCPs. 6. Factors that predicted social workers’ involvement with the HCP included age, attitudes, perceptions of barriers, and perceived physician support.
Lacey, 2006 [42] (USA)	To describe nursing home social services staff roles and perceptions related to EoL medical decision making for nursing home residents in end-stage dementia.	A cross-sectional survey	A convenient sample of nursing home social workers.	138	1. 97% identified themselves as being responsible for discussing ADs on admission. 2. 90% said that they often or always provided written information to newly admitted residents and family members (a PSDA requirement). 3. 72% said that they helped families clarify their thoughts about LST choices. 4. 45% agreed or strongly agreed that social work discipline is best suited to discussing ADs with residents and family members. 5. A substantial proportion of respondents (1–75%) did not correctly answered relevant “true/false” questions about LST.
Black, 2005a [40] (USA)	To examine social workers’ personal death attitudes and experiences in relation to their ADs communication practice behavior.	A cross-sectional survey	A cluster sample of social workers employed at 6 hospitals (response rate: 94%)	29	1. 38% of the respondents reported recent experience with terminal illness; 48% reported recent death of a close friend or a family member. 2. Social workers with either fearful or avoidant death attitudes collaborated less frequently with other professionals about ADs, compared to practitioners with neutral death attitudes. 3. Social workers reporting recent personal experiences with terminal illness differed from practitioners without personal experiences by less frequent collaboration with others, initiation of the topic of AD, and disclosure of information regarding AD.
Lacey, 2005 [43] (USA)	To discuss the responses of nursing home social workers regarding their perceived use of skills related to ACP for nursing home residents.	A cross-sectional survey	A convenient sample of nursing home social workers.	138	1. 93% of the respondents said they often or always educate families about ADs. 2. 93% said they are often or always involved in care planning for residents with dementia. 3. 77% said they often or always were involved in conflict resolution with families. 4. 58% reported often or always educate staff about ADs. 5. 55% reported they often or always engaged in developing a more professional role for social workers in their respective facilities.
Black, 2004 [41] (USA)	To describe social workers’ AD communication practices with hospitalized elderly patients	A cross-sectional survey	A cluster sample of social workers employed at 6 hospitals (response rate: 94%)	29	1. Social workers frequently and comprehensively address the phases of the AD communication process in their practices with hospitalized elderly patients. 2. 52% of the social workers reported spending between 0.5 h and one hour daily in their AD communication practices with hospitalized elderly patients. 3. While 19% of the social workers believed that they were spending a sufficient amount of time discussing ADs, 82% reported that the time they spent in AD communications was inadequate.
Csikai et al., 2004 [25] (USA)	To identify ethical problems in EoL care decision making faced by oncology social workers	Qualitative interviews	Hospital social workers	12	1. Common ethical issues identified through thematic analysis included preservation of patients’ autonomy/self-determination, beneficence of health care providers, and medical futility of end-of-life treatments. 2. Continued communication with all parties involved was key in resolving ethical problems. Referrals to ethics committees or ethics consultation teams occurred for more complex cases. 3. Although they viewed the development of relevant guidelines as desirable, many indicated there would be numerous barriers to their implementation.
Heyman & Gutheil, 2003 [36] (USA)	To examine the attitudes of entry-level MSW students toward end-of-life planning and the factors associated with these attitudes.	A cross-sectional survey	A cluster sample of MSW students (response rate: 64%)	267	1. 57% of the respondents were knowledgeable about living wills. 2. Attitudes toward end-of-life planning was significantly correlated with age, knowledge, personal comfort with end-of-life discussions, and personal desire for treatment.
Werner & Carmel, 2001 [22] (Israel)	to examine the involvement, beliefs, and knowledge of social workers in health care settings in the process of making decisions regarding LSTs.	A cross-sectional survey	A convenience sample of social workers	68	1. 31.2% of the social workers (in Israel) reported never or almost never being involved in asking patients about their wishes. 2. Social workers’ involvement in decision making regarding LST were negatively associated with their perceptions regarding physicians’ involvement and positively associated with higher exposure to terminal patients. 3. Most participants (52 to 71%) agreed with different statements regarding social workers’ responsibility for talking with patients and participating in the process of decision making regarding LSTs. 4. The area in which social workers reported being more involved were activities related to family members. The extent of social workers’ involvement was related to their beliefs regarding their role and their knowledge about LSTs.
Baker, 2000 [37] (USA)	To describe the knowledge and attitudes of health care social workers regarding ADs.	A cross-sectional survey	A systematic random sample of social workers (response rate: 65%)	324	1. 98% of the respondents had positive attitudes regarding the use of ADs. 2. 82% had a high to moderate level of knowledge about ADs. 3. Those with more experience working with the elderly had higher levels of knowledge. 4. Those employed in nursing homes and hospice settings had more positive attitudes than did those working in other health care facilities.

*ACP* advance care planning, *AD* advance directive, *EoL* end-of-life, *HCP* health care proxy, *NASW* National Association of Social Workers, *POLST* Physician’s Order for Life Sustaining Treatment, *PSDA* Patient Self-Determination Act, *SD* standard deviation

**Table 2:** Summary of descriptive studies that included both healthcare and social care professionals

Study (country)	Objectives	Design	Participants	N	Major findings
Yee et al., 2011 [21] (Singapore)	To explore the knowledge, attitudes and experience of renal health-care professionals in Singapore on ACP for patients with end-stage renal failure.	A cross-sectional survey	All renal physicians, renal nurses, renal medical social workers (MSWs) and other allied health professionals working in Singapore (response rate: 90.6%)	562	1. MSWs and physicians had higher knowledge scores than nurses and others. 2. 82.4% of doctors and 100% of MSWs considered ACP discussions as part of their role, but only 37.1% of nurses and 38.1% of other allied health professionals thought likewise. 3. MSWs appeared to be the most confident in conducting ACP discussions. Nurses were the least confident, and most were fearful of upsetting patients and families. 4. 90% of the nurses and 71.4% of others occasionally or never discussed ACP with their patients compared to 66.6% of physicians and 53.9% of MSW. Of those who discussed ACP with their patients, 82% of the physicians and 90% of MSW initiated the discussions themselves compared to nurses (18.6%).
Heyman, 2008 [45] (USA)	To examine the factors associated with health care professionals’ attitudes toward the HCP, one form of an AD.	A cross-sectional survey	A random sample of nurses and social workers who were members of relevant professional associations (response rate: 34% for nurses and 46% for social workers)	213	1. Social workers and nurses had generally positive attitudes toward the HCP, but social workers had higher attitude scores than nurses. 2. Factors that predicted attitudes included profession and training in end-of-life care. 3. Professionals’ perception of individual/family barriers and their perception of system barriers increase, their attitude towards HCP decreases. 4. Health care professionals who had prior training in EoL planning had more positive attitudes toward the HCP than those who did not have training. 5. The top three perceived barriers against HCP were: patient discomfort in discussing the topic; patient knowledge about the HCP; and patient’s fear of death.
Laje et al., 2007 [48] (USA)	To assess nursing home physicians’ and social workers’ perceptions of a patient plan of care form	A cross-sectional survey	A convenient sample of physicians and social workers	37 physicians & 60 social workers	1. 85.6% of the respondents stated that social workers are completing the form, while 49% of the physicians and 25% of the social workers said that physicians are involved in completing the form. 2. 92.5% of the respondents stated that the patient plan of care from was completed within less than 2 weeks of admission.
Black, 2006 [26] (USA)	To explore differences in AD communication practices by comparing and contrasting nurses’ and social workers’ perceptions of their roles.	Qualitative interviews	A purposive sample of care professionals from a moderate-sized facility	6 nurses & 5 social workers	1. Nurses’ communication tended to focus on pragmatic information, but social workers expressed addressing the “meaning” of the choices faced by patients. 2. For nurses, ADs were addressed routinely with almost all of their patients through institutional admission procedures. Social workers’ AD communication practices were limited to the patients encountered through screening or referrals. 3. Social workers indicated an awareness of their roles as advocates in promoting the content of the patient’s wishes with family members as well as with other health care providers. Nurses reported advocating on the patient’s behalf with other providers. 4. When discussing the appointment of a surrogate decision maker, nurses reported urging patients to choose a family member and social workers reported encouraging patients to question the selection of a family member as a surrogate. 5. Nurses perceived particular expertise among social workers as good communicators, and social workers acknowledged nurses’ primary role with patients.
Black, 2005b [46] (USA)	To examine the roles and AD communication practices of social workers as members of the interdisciplinary health care team.	A cross-sectional survey	A cluster sample of multi-disciplinary professionals employed at 6 hospitals	32 physicians, 74 nurses, 29 social workers	1. Physicians reported the lowest frequency of initiating the AD communication while social workers reported the highest. 2. Compared to both nurses and physicians, social workers disclosed more information about the purpose of ADs, patient rights to formulate or modify the documents, parameters about specific ADs, and the need to document patient ADs in the medical record. 3. Compared to both nurses and physicians, social workers more frequently talked with patients about potential proxy choices, assessed prospective proxy’s capacity to serve as a surrogate, and confirmed that identified proxy was willing, able, and available to serve. 4. Social workers discuss the treatment options of feeding tubes, respirators, and comfort measures more than physicians and nurses, and the option of hospice more than nurses. 5. Compared to both nurses and physicians, social workers more frequently urged patients to think about their values in living as they consider the impact of potential treatment options. 6. Social workers also differed from physicians and nurses by interacting more frequently with family and others via the request of another health care professional. 7. 52% of the social workers spent 0.5–1.0 h daily in their AD communication practices, while the physicians (90%) and nurses (86%) spent less than 0.5 h daily for AD communication.
Werner et al., 2004 [23] (Israel)	To examine nurses’ and social workers’ attitudes and beliefs about and involvement in LST decisions.	A cross-sectional survey	A cluster sample of nurses who were working in 3 large medical centers and a convenient sample of social workers from different health care settings.	274	1. Whereas nurses reported being more involved in the daily care of terminally ill patients, social workers reported being more involved in discussions with patients and family members. 2. Social workers reported consistently stronger beliefs than nurses regarding their role in the decision-making process and their role with patients and family members. 3. Nurses were more willing than social workers to use artificial feeding and less willing to use mechanical ventilation and CPR for all conditions.
Neuman & Wade, 1999 [47] (USA)	To explore the perceptions of health care providers as to how effective AD arrangements were in assuring compliance with the patients’ wishes, and their satisfaction levels with the process.	A cross-sectional survey	An interdisciplinary sample of health care providers practicing in a variety of settings (response rate: 33%)	116	1. 64% of the respondents indicated that they had direct responsibility in overseeing the facility’s ADs program or in discussing ADs with patients’ families. 2. Social workers as a group reported lower levels of satisfaction with AD laws and systems than nurses and other professionals. 3. Respondents working in hospitals were more likely to report encountering patient and family conflict regarding treatment decisions, difficulty in communicating with the patient and family and lack of adequate guidance from the medical staff.

*ACP* advance care planning, *AD* advance directive, *CPR* cardiopulmonary resuscitation, *HCP* health care proxy, *LST* life-sustaining treatment, *MSW* medical social worker

**Table 3:** Summary of interventional studies

Study (country)	Objectives	Design	Participants	N	Major findings
Song et al., 2010 [31] (USA)	To determine whether homeless persons will complete a counselling session on ACP with a social worker and fill out a legal AD.	A single-blind, randomized controlled trial	Homeless persons recruited from 8 sites were randomly assigned to one of 2 groups: a self-guided intervention and a counselor-guided intervention	262	1. The overall completion rate for ADs was significantly higher in the one-to-one counselling group than in the self-guided group (37.9% vs. 12.8%). 2. This difference persisted across all of the eight sites and most subgroups.
Johnson & Stadel, 2007 [27] (USA)	To test the efficacy of a preadmission educational interview by a social worker on the completion of Ads	A quasi-experimental study	Adult orthopedic surgical patients who were admitted to a hospital for hip or knee replacement surgery	54	1. After the intervention, 43% of patients in the treatment group had a health care proxy on their charts, compared to 6% of those in the comparison group (*p* < .005). 2. Age, residence, ethnicity, and diagnosis were found not to have significant impact on signing a health care proxy.
Pearlman et al., 2005 [28] (USA)	To increase ACP use through an educational and motivational intervention by social workers.	A randomized controlled clinical trial	A sample of veteran patients recruited from 23 providers were randomized into the intervention or control group.	280	1. Compared to the controls, the intervention patients reported more ACP discussions with their providers (64% vs 38%). Living wills were filed in the medical record twice as often in the intervention group (48% vs 23%). 2. Provider-patient dyads in the intervention group had higher agreement scores than the control group for treatment preferences, values, and personal beliefs (*p* < .01).
Morrison et al., 2005 [29] (USA)	To assess the effect of a ACP intervention directed at social workers on identification and documentation of preferences for medical treatments and on patient outcomes	A randomized controlled trial	Newly admitted long-term care residents were allocated into either an intervention or a control group.	139	1. Intervention residents were significantly more likely than residents in the control group to have their preferences regarding cardiopulmonary resuscitation (*p* = .005), artificial nutrition and hydration (*p* < .01), intravenous antibiotics (*p* < .01), and hospitalization (*p* < .01) documented in the nursing home chart. 2. Control residents were significantly more likely than intervention residents to receive treatments discordant with their prior stated wishes.
Dipko et al., 2003 [32] (USA)	To examine the effectiveness of group education sessions in increasing completion of ADs	A retrospective cohort control study	A cohort of outpatients was divided into three categories: group participants, individually educated patients, and the patients with no AD education.	13,913	1. Social work education of any kind resulted in an overall completion rate of 20% versus 2.1% in the non-intervention group. 2. Group education was twice as effective as an individual social work session, and as effective as multiple sessions, but less time consuming.
Gockel et al., 1998 [30] (USA)	To evaluate the effect of an educational intervention by social workers on the completion of ADs	An uncontrolled observational study	A convenient sample of outpatients recruited at an ambulatory care setting	203	1. An educational intervention increases the percentage of individuals who initiate an AD. 2. Patients with more hospitalizations were more likely not to have an AD.
Bailly & DePoy, 1995 [33] (USA)	To evaluate a social work program designed to promote older people’s autonomous decision making regarding ADs.	An uncontrolled observational study	A convenient sample of older clients who regularly used family medical care services.	10	1. The results revealed a continuum of willingness among elderly people to address future decision making.
Luptak & Boult, 1994 [34] (USA)	To examine the effectiveness of an intervention implemented by a social worker to help frail elders to record AD	An uncontrolled observational study	All patients who visited an experimental geriatric evaluation and management clinic during a period of 14 months.	34	1. 71% of the participants recorded AD. Of these, 96% named a proxy and 83% recorded specific treatment preferences.

*ACP* advance care planning; AD: advance directive

Research themes emerged in these studies varied greatly, including social workers’ awareness of, knowledge about, attitudes toward, and involvement in ACP, ACP communication, beliefs and decision making about life-sustaining treatments, ethical issues related to EoL decision making, and the effect of social work intervention on ACP engagement. The findings of these studies could be classified into six categories (Table 4).

**Table 4:** Summary of research themes in the included studies

Themes	Major findings
Attitudes toward the use of ACP	• 98% of social workers had positive attitudes regarding the use of ADs [37]. • 97% of social workers identified themselves as being responsible for discussing ADs on admission [42]. • 62% of social workers stated they had a role in EoL planning [39]. • 45% of social workers agreed that social work discipline is best suited to discussing ADs with residents and family members [42]. • 52–71% of social workers agreed that they are responsible for talking with patients and participating in the process of decision making regarding LSTs [22]. • 72% of social work students in the US indicated that they felt comfortable discussing the topic of death [11]. • 41% of social workers in South Korea felt comfortable with death discussion [19]. • Social workers and nurses had generally positive attitudes toward the HCP, but social workers had higher attitude scores than nurses [45]. • Social workers in health and aging had significantly higher positive attitude scores than those not in health and aging [35]. • Those employed in nursing homes and hospice settings had more positive attitudes than did those working in other health care facilities [37].
Knowledge, education & training regarding ACP	• 82% of social workers had a high to moderate level of knowledge about ADs; those with more experience working with the elderly had higher levels of knowledge [37]. • 57% of social work students were knowledgeable about living wills [36]. • For health care social workers, 88.8% had training in EoL care issues, compared with 53.2% in other social workers, and 78.3% indicated that they received EoL care training in continuing education programs [35]. • 60% of social worker assistants said that they receive no training regarding ADs, although 53% said they have recently received training on the POLST [24]. • A substantial proportion of respondents (1–75%) did not correctly answered relevant “true/false” questions about LST [42]. • Social workers as a group reported lower levels of satisfaction with AD laws and systems than nurses and other professionals [47]. • 73% of social workers in South Korea reported no knowledge of ADs [19].
Involvement in ACP	Findings related to ADs • 96% of the respondents reported that social workers in their department are conducting ACP discussions with patients/families [44]. • 80% of the respondents reported that social workers are responsible for educating patients and/or families about ACP options [44]. • 93% of social workers said they often or always educate families about ADs [43]. • 68% of the respondents reported that social workers are responsible for documenting ACP [44]. • 85.6% of the respondents stated that social workers are completing the care plan form, while 49% of the physicians and 25% of the social workers said that physicians are involved in completing the care plan form [48]. • 90% of social workers said that they often or always provided written information regarding ADs to newly admitted residents and family members (a PSDA requirement) [42]. • 93% of social workers said they are often or always involved in care planning for residents with dementia [43]. • One third of social workers stated that they initiate the AD conversation, 33% said that nurses initiate the AD conversation, and others said that AD conversation is done at admission or during care planning meeting [24]. • 73% of social worker assistants stated that they have conversations about AD during care meetings [24]. • 31.2% of social workers reported never or almost never being involved in asking patients about their wishes [22]. • 40% of social workers indicated that they provide a facility-specific form to residents regarding ADs, and additional 40% noted that no AD forms are provided at all [24]. • 55.3% of the social workers (in South Korea) had experience aiding elderly patients and/or family members by providing information about ADs [20]. • 83.3% of the social workers (in South Korea) reported that they did not have any experience working with terminally ill patients; only 2.8% reported that they had ever provided AD planning [19]. • The area in which social workers reported being more involved was activities related to family members [22]. • 64% of social workers indicated that they had direct responsibility in discussing ADs with patients’ families [47]. • 52% of social workers reported spending 0.5-1 h daily in their AD communication practices with hospitalized elderly patients and their families, and 82% of them reported that the time they spent in AD communications was inadequate [41]. • Compared to physicians and nurses, social workers spent more time daily in their AD communication practices with hospitalized elderly patients [46]. Findings related to HCP • 72.3% of health care social workers had completed a HCP, compared with 48.8% of other social workers [35]. • 44% of social workers stated they are very often involved in discussing the HCP with clients, and 37.3% are very often involved in counseling clients regarding HCPs [39]. • Almost 25% of social workers stated that they were very often involved in receiving requests from other professionals to explain HCP, and 23.8% stated they were very often involved in completing HCPs [39].
Social workers’ roles in interdisciplinary health care teams	• 100% of MSWs and 82.4% of doctors considered ACP discussions as part of their role, but only 37.1% of nurses thought likewise [21]. • 72.5% of social workers rated education as an important role and 31.5% said that facilitating decision making was an important role for them in EoL planning [39] • In Singapore, 53.9% of MSWs occasionally or never discussed ACP with their patients compared to 66.6% of physicians and 90% of the nurses. Of those who discussed ACP with their patients, 90% of MSWs and 82% of the physicians initiated the discussions themselves compared to 18.6% of nurses [21]. • Social workers reported consistently stronger beliefs than nurses regarding their role with patients and family members in the decision-making process [23]. • Compared with physicians and nurses, MSWs appeared to be the most confident in conducting ACP discussions [21]. • Nurses acknowledged particular expertise among social workers as good communicators, and social workers perceived nurses’ primary role with patient [26]. • Social workers reported being more involved in discussions with patients and family members, whereas nurses reported being more involved in the daily care of terminally ill patients [23]. • For nurses, ADs were addressed routinely with almost all of their patients through institutional admission procedures and routine nursing care. Social workers’ AD communication practices were limited to patients encountered through screening or referrals that typically did not originate for ACP purposes [26]. • Social workers indicated an awareness of their roles as advocates in promoting the content of the patient’s wishes with family members and health care providers [26].
Ethical issues related to the use of ACP	• 97% of social worker students indicated that self-determination is a very important principle in social work practice [11]. • 75% of social worker students answered that they would be troubled if problems of self-determination resulted in conflicts [11]. • 77% of social workers said they often or always were involved in conflict resolution with families [43]. • Nearly all respondents indicated a situation they had once dealt with where the family requests something different from what is written on the patient’s ADs [24]. • 72% of social workers reported that they helped families clarify their thoughts about LST choices [42]. • Common ethical issues identified through thematic analysis included preservation of patients’ autonomy/self-determination, beneficence of health care providers, and medical futility of end-of-life treatments [25]. • Social workers working in hospitals were more likely to report encountering patient and family conflict regarding treatment decisions, difficulty in communicating with the patient and family and lack of adequate guidance from the medical staff [47]. • Continued communication with all parties involved was key in resolving ethical problems [25].

*ACP* advance care planning, *AD* advance directive, *HCP* health care proxy, *LST* life-sustaining treatment, *MSWs* medical social workers, *POLST* physician’s order for life-sustaining treatment

### 1. Social workers’ perceptions and attitudes toward ACP

Most social workers had positive attitudes toward ACP, which varied across studies or with particular tasks. Social workers who were working in health and aging areas were more likely to have positive attitudes than those working in other areas. The attitudes toward ACP were significantly correlated with age, knowledge, personal comfort with death discussions, personal value regarding self-determination, and personal desire/preference for relevant treatments [11, 19, 36]. The attitudes toward HCP were associated with their perception of individual/family barriers, perception of system barriers, and training in EoL planning [45]. Compared with those in the US, fewer social workers in South Korea felt comfortable with death discussion [11, 19].

### 2. Social workers’ knowledge, education, and training regarding ADs or ACP

Most social workers in the US had good knowledge about ADs, especially among those with more experience working with the elderly or in health care area [35–37]. A study indicated that 89% of health care social workers had training in EoL care issues [35], but another study reported that 60% of social workers in nursing homes received no training regarding ADs [24]. Usually, they received EoL care training in continuing education programs [35]. Their knowledge regarding life-sustaining treatments seemed to be insufficient [42]. Compared with nurses and physicians, fewer social workers reported satisfaction with AD laws and systems [47]. Unlike those in the US, most social workers in South Korea didn’t receive education or training regarding ACP [19].

### 3. Social workers’ involvement in ACP discussion

An earlier study indicated that 31% of social workers in the US were never or almost never involved in asking patients about their wishes [22], but later studies suggested that over 90% of health care social workers were involved in ACP practice, mainly at admission to care settings or in long-term care facilities [42–44], even though only one-third of social workers stated that they initiated ACP conversations [24].The majority of social workers (73%) had conversations about ADs during care meetings [24]. They were often involved in educating patients and/or families about ACP options, providing information about ACP, and documenting ADs [20, 42–44, 48]. A facility-specific form regarding ADs were routinely provided to the residents in 40% of nursing facilities [24]. Around 37–44% of social workers were often involved in discussing the HCP with clients [39]. Almost 25% of social workers were often involved in receiving requests from other professionals to explain HCP [39]. The area in which social workers reported being more involved was activities related to family members [22]. Over 64% of social workers indicated that they had direct responsibility in discussing ADs with patients’ families [47]. Compared to physicians and nurses, social workers spent more time daily in their AD communication practice with patients, but most of them reported that the time they spent in AD communications was inadequate [41, 46]. Unlike those in the US, many social workers in South Korea did not have any experience working with dying patients for ACP [19, 20].

Social workers’ involvement in ACP or EoL decision making was correlated with their age, attitudes, perceptions of barriers, perceived physician support [39], and perceptions regarding physicians’ involvement [22], fearful/avoidant death attitudes [38, 40], personal experiences with terminal illness [40], peace of mind, and control over decisions [38]. Those with one or more of these problems collaborated less frequently with other professionals about ADs and were less frequently to initiate the topic or disclose information regarding ACP [40]. Social workers with higher exposure to terminal patients was more likely involved in decision making regarding LST [22]. Major barriers against the completion of ADs included clients’ discomfort with the topic, clients’ knowledge about ADs, clients’ fear, timing of discussions, and clients’ belief about control over their lives [39].

### 4. Social workers’ perceptions of their roles

Compared with physicians and nurses, medical social workers reported stronger beliefs regarding their role with patients and family members in the decision-making process [23]; they appeared to be the most confident in conducting ACP discussions [21]. In a study, 100% of medical social workers considered ACP discussion as part of their role, but only 37.1% of nurses and 82.4% of doctors thought likewise [21]. Most social workers (72.5%) rated education as an important role and 31.5% said that facilitating decision making was an important role for them in EoL planning [39]. Usually, social workers discussed ACP with their patients more frequently than physicians and nurses [21]. A study noted that social workers reported the highest frequency of initiating the topics about ADs while physicians reported the lowest [46]. Compared with physicians and nurses, social workers more frequently discussed the options of LST and the option of hospice, and more frequently talked with patients about potential proxy choices [46]. They also differed from physicians and nurses by interacting more frequently with families and others [46]. Nurses acknowledged particular expertise among social workers as good communicators, whilst social workers perceived nurses’ primary role with patients [26]. For nurses, ADs were addressed routinely with almost all of their patients through institutional admission procedures and routine nursing care. Social workers’ AD communication practices were mainly limited to the patients encountered through referrals or screening that typically did not originate for ACP [26].

### 5. Ethical issues related to EoL decision making

Most social workers considered self-determination as a very important principle, and agreed that they would be troubled if problems of self-determination resulted in conflicts [11]. More than three fourths of social workers were often or always involved in conflict resolution with families [43]. A study reported that nearly all of the social workers indicated a situation they had once dealt with, where the family requests something different from what was written on the patient’s ADs [24]. A majority of social workers (72%) reported that they had helped families clarify their thoughts about LST choices [42]. Common ethical issues related to ACP practice included preservation of patients’ autonomy/self-determination, beneficence of health care providers, and medical futility of end-of-life treatments [25]. Social workers working in hospitals were more likely to report encountering patient and family conflict regarding treatment decisions, difficulty in communicating with the patient and family, and lack of adequate guidance from medical professionals [47]. Continued communication with all parties involved was key in resolving ethical problems [25].

### 6. Effectiveness of social work intervention on ACP engagement

It was reported that social workers’ involvement in ACP increased the rate of patients’ ACP discussions with care providers [28], patients’ documentation of their living wills or ADs in the medical record [28] or in the nursing home chart [29], completion rate for ADs [31, 32, 34], and the appointment of HCPs [27, 34]. Compared to controls, patients in the intervention groups had higher agreement scores for treatment preferences, values and personal beliefs [28], and were less likely to receive treatments discordant with their previously expressed wishes [29]. Age, residence, ethnicity, and diagnosis didn’t have significant impact on signing a HCP [27].

## Discussion

In this review, empirical or scientific findings relevant to social work practice of ACP were systematically examined and thematically synthesized. A total of thirty one studies were included. Our results provided a whole profile of social workers’ attitudes toward, knowledge of, and involvement in ACP practice. The findings suggest that there is a consensus among social workers that ACP is their duty and responsibility and that social workers play an important role in promoting and implementing ACP through an array of duties such as initiating ACP discussions, advocating patients’ rights, patient and family education or counseling, facilitating communication and conflict resolution, as well as documenting discussions or ADs.

It should be noted that most of the included studies were conducted in the US, where 45–47% of all deaths occurred in hospitals and additional 22% (28% for those aged over 65 years) occurred in residential care facilities in the years 2003–2005 [49]. For nursing home residents, up to two-thirds of them died in place [43, 50]. In 2015, there were 155,500 healthcare social workers in the US [51]. Since 1991, following the passage of the Patient Self-Determination Act, health practitioners including social workers in different organizations such as hospitals, hospices and nursing homes were mandated to inform their adult patients about their rights in making EOL care related decisions and formulating living wills or ADs [11, 52]. Given the fact that many social workers felt inadequately prepared for work in the field of practice with dying and bereaved patients [53], a national program was initiated to promote professional growth among social work leaders in the late 1990s, and various continuing education and certificate programs emerged for social work practitioners thereafter [5]. Following these programs, social work professionals in the US have made considerable progress toward improving ACP practice, as indicated by the results of the studies included in this review. However, lack of knowledge regarding ACP and insufficient training or education among social workers are evident in Asia countries such as South Korea [19].

Unlike in the US, social workers’ roles and responsibilities in the UK are different due to the disparity in the structures of the national healthcare and social care systems and variation in the professional systems between the two countries. The introduction of advance communication related to EoL care was formalized in England and Wales through the Mental Capacity Act 2005, which became effective in 2007 [54]. In the US, social work practice focuses more often on the approaches of psychosocial intervention, whereas social workers’ roles in the UK are prescribed and limited to safeguarding, assessment, care planning, empowerment and partnerships [54]. To date, empirical studies of social workers’ involvement in ACP are still rare in European countries.

Of the included studies, there were large variations in the concepts of ACP and ADs. For different concepts, their contents and meaning may be significantly different. These variations make it difficult to collate and compare research results across studies. Usually, ACP is viewed as a process to clarify values, wishes, preferences, and goals regarding care. This process may not be completed following one session of discussion. It may take a period of time and include many sessions of communication or discussion. Emanuel et al. (1995) proposed five steps for an idealized process of ACP: raising the awareness, facilitating a systematic discussion, completing ADs, reviewing ADs periodically, and applying ADs in actual circumstances [55]. Black (2000) operationalized the process into 7 phases: initiation of the topic, disclosure of information, identification of a surrogate decision-maker, discussion of treatment options, elicitation of patient values, interaction with family members, and collaboration with other care professionals. Empirical studies suggested that social workers were involved in each step and phase of ACP [40, 41, 46]. ADs or living wills are legal evidence of one’s preferences regarding medical interventions at the end of life. Very often, a dying patient needs to decide whether or not to refuse a specific type of invasive life-sustaining treatment. If a patient wishes to make an AD to refuse a treatment, the patient may need to discuss this with a health care professional who is fully aware of his/her medical conditions and cure options as well as associated problems, and the AD must be in writing, signed and witnessed. In such a situation, roles between social worker and other health professional overlap, which may lead to confusion. As indicated in a study, most social workers do not have sufficient knowledge of life-sustaining treatments [42]. Although social workers may not be the right persons for documenting patients’ ADs in such a situation, they can act as an educator, counselor, context interpreter, advocator, and team member [3, 56]. Thus, it is understandable that social workers usually spend more time than physicians and nurses in their daily ACP practice with patients, but are more likely to feel that the time spent in ACP communications is inadequate [41, 46]. Apart from decision-making regarding life-sustaining treatment, ACP includes several other important elements such as health care proxy, preferred priorities of care, and preferred place of care. Social workers are the experts to communicate with patients and their families about these issues. Social workers can also use structured ACP tools to facilitate ACP discussions [54].

Among the included studies, there is no consensus about the time and manner to initiate ACP communications. A qualitative study indicates that social work involvement occurs most often at the request of other staff, specifically nurses [50]. Some studies suggest that there are key transitions in the disease courses whereby ACP may be particularly needed, such as hospital or nursing home admissions [16]. Sometimes, social workers may need to gain permission from a patient’s physician to engage in ACP communications. Very often, the dying persons are heavily constrained in their exercise of autonomy, choice and control [4]. Moreover, different countries may have different policies or approaches to promote ACP/ADs. For example, ACP is widely promoted among healthy adults of the public in the US [57], whereas an official guideline in the UK has cautioned against a rigid and prescriptive approach in order to avoid harm to relevant persons, because the discussion my cause distress [58]. Thus, it is important for social workers to know when and how to initiate ACP communications within a particular socio-cultural context. The influence of culture on EoL care preferences has been documented elsewhere [59]. There is also evidence that clinical social work practice of EoL care in a Western form, which stresses open discussion of impending death and individualized counselling, does not meet the needs of people with different cultural backgrounds or in different nations in responding to dying and death [7].

Some of the included studies suggested insufficient knowledge of ACP among social workers and insufficient education of social work students, which might have hindered them in implementing ACP [6, 42]. Many social workers indicated that much of their knowledge of ACP and ADs come from continuing education programs [42], mainly due to the lack of EoL care content in social work textbooks and the absence of faculty trained to teach EoL care [5]. While numerous publications and books in the field of EoL care have been available, social work textbooks provide little content on EOL care in general and ACP in particular [5]. While there are courses of death and dying, there is little clinical supervision focusing on EoL care and ACP practice [50]. The lack of curricular content about ACP in educational social work programs may result in practitioners being ill-equipped to work effectively in ACP practice. Nowadays, these situations may be improved in the US, but remain to be great challenges for social work professionals in other countries where EoL care has not been included social work practice agenda until recent years. Apart from educational resources, some other factors associated with social workers’ attitudes toward, knowledge of, and involvement in ACP practice, as summarized in this review, should be taken into account when promoting ACP from a social work perspective.

There are several limitations in this review. First, similar to any other systematic reviews, the keywords we employed may not have captured all relevant studies; some potentially relevant articles that are only available in other databases or in other languages may have been neglected. Second, study quality was not ranked for the included studies due to heterogeneity in study designs. Interpretation and generalization of the results should be cautious due to small sample size and non-representative sample in some of the included studies. Third, most of the included studies were cross-sectional surveys conducted in the US, which may limit our understanding social work practice of ACP in European and other countries such as Australia, where ACP medico-legalities and social work scopes of practices are different from that in the US. Lastly, the differences in social work practice of ACP communications at different care settings or for different life-limiting illnesses were not differentiated due to insufficient information. Further studies in these aspects would be meaningful. Despite these limitations, our review is the first to systematically and thematically summarize relevant findings in the field, which may have implications for professional social work education, EoL care practice, and healthcare or social care policy making.

## Conclusion

On the basis of available evidence, this review provides a whole profile of social workers’ attitudes toward, knowledge of, and involvement in ACP practice, mainly in the US. The findings suggest that social workers can be core members of health care teams providing EoL care, and that social workers play an important role in promoting and implementing ACP. This review provides useful information or knowledge for implementing ACP through illustrating social workers’ perspectives and experiences. It also suggests insufficient knowledge and limited education regarding ACP among social workers, which may be major barriers for social work practice in this field. The results of this review can assist social workers, professionals, educators, and policy makers to develop policies, programs, and practical guidelines for ACP-related education and practice so as to create an appropriate environment for promoting ACP and increase the competency of social workers in EoL care practice. Where ACP is clearly legislated as an act that must be advocated for dying persons, more attention should be focused on logistics of clinical practice of ACP communications. Provision of ACP-related curriculum, educational programs and practical information for social workers, especially those in the countries or regions where EoL care services are underdeveloped, is recommended so as to increase their knowledge about ACP and conversation skills. Further research is also warranted to understand the complexity inherent in social work practice of ACP discussions or communications in formulating EOL care preferences at different care settings for different life-limiting illnesses within different socio-cultural contexts, so as to promote quality of life and well-being of dying persons and their families.

## Abbreviations

ACP: Advance care planning
ADs: Advance directives
EoL: End-of-life
HCP: Health care proxy
LST: Life-sustaining treatment
MSWs: Medical social workers
POLST: Physician’s order for life-sustaining treatment
PSDA: Patient Self-Determination Act
UK: United Kingdom
US: United States
